# Clinic Models for Care Delivery in CKD: How Should We Define Success?

**DOI:** 10.34067/KID.0000000795

**Published:** 2025-06-26

**Authors:** Mark Canney, Swapnil Hiremath

**Affiliations:** Department of Medicine, University of Ottawa, Ottawa, Ontario, Canada and Ottawa Hospital Research Institute, Ottawa, Ontario, Canada

**Keywords:** CKD

There are many facets to providing care for patients with CKD. Guidelines are awash with recommendations for laboratory test monitoring and target values; there are established risk prediction tools to guide decision making about timing of referral for dialysis access or kidney transplantation, and there are new and emerging treatment options to slow disease progression that require implementation in the clinic.^1,2^ These aspects of care lend themselves well to policies and protocols within a structured clinic model. In addition, there are opportunities to improve the health of someone living with CKD, whether that is through lifestyle interventions, optimizing nutrition, reducing polypharmacy, empowering them through education, or providing psychosocial support. Even the most committed of physicians would struggle to hit all of these targets in a single clinical encounter. The inclusion of specialized nurses, nutritionists, pharmacists, and social workers in a diverse care team has the potential to greatly increase the scope of what can be achieved in a clinic visit.^3^ The desire to provide this level of holistic care has culminated in the development of multidisciplinary CKD clinics. Although grounded in the same underlying principles, these clinics have different models of care delivery and varying composition of team members depending on local availability and expertise.

In this issue of *Kidney360*, Mateo Chavez *et al.* evaluated clinical outcomes associated with the introduction of an interdisciplinary CKD clinic supported by a clinic registry.^4^ Based in the Mayo Clinic, the team is led by nurse practitioners and includes nurse educators, dieticians, social workers, and a capacity coach. Patients with additional complexity may have their care comanaged with a nephrologist or fully managed by a nephrologist. Using a retrospective cohort of 534 patients enrolled in the clinic between March 5, 2021, and May 31, 2022, the investigators used a pre/poststudy design to evaluate the frequency of health care utilization and the progression of CKD. Compared with the preclinic period, there was a 26% decrease in the frequency of hospital admissions (incidence rate ratio, 0.74; 95% confidence interval, 0.60 to 0.91) and a 30% decrease in the frequency of emergency department visits (incidence rate ratio, 0.70; 95% confidence interval, 0.57 to 0.87) after the introduction of the interdisciplinary clinic. After entering the clinic, there was variable movement of patients in and out of CKD stages. In an exploratory analysis, patients who exercised <5 days per week and patients who were unemployed or had a disability were more likely to experience CKD progression.

One has to be cautious when interpreting the “effect” of a complex intervention such as a multidisciplinary clinic team, and there are important aspects of study design to consider when evaluating outcomes. The first issue is patient selection. In the Mayo Clinic model, patients could be referred at any stage of CKD without other eligibility criteria, as long as they were willing to attend clinic visits. This could arguably introduce a selection bias because motivated patients are perhaps more likely to seek referral and attend clinic visits. The median eGFR at clinic entry was 26 ml/min, and 40% of patients had an eGFR of 30 ml/min or higher. It has been previously shown that the probability of regression (improvement in GFR to a better CKD stage) is similar to the probability of progression (worsening of GFR to a worse CKD stage) among older adults, even those with stage 4 CKD.^5^ It was therefore not surprising that most patients remained stable in the cohort with limited movement in or out of CKD stages during follow-up. The second issue is the choice of index date for capture of clinical events. By the time patients are referred to a multidisciplinary clinic, there has already been substantial heterogeneity in their experiences of kidney disease. Some patients, such as those with congenital kidney disease or GN, may have been living with CKD for decades, whereas other patients may have only recently been diagnosed with CKD. When the index date is defined as the first date of clinic entry, this variability in exposure to CKD is lost. Furthermore, patients who have experienced a rapid decline in kidney function may have had no opportunity to avail of the clinic because they already experienced CKD progression. This concept is termed “depletion of susceptibles” and can result in a biased sample under study because high-risk patients are excluded from the analysis.^6^ In this study, the investigators used a pre/postdesign to investigate the frequency of health care utilization for 6 months before and after clinic implementation. By including patients at clinic entry and then going back in time to evaluate clinical outcomes, the analysis is also susceptible to immortal time bias because future information is being used about a patient to define their exposure status. The magnitude of bias was probably small in this case because of the relatively short observation period. The third issue is unmeasured confounding. As with all retrospective studies, the investigators were limited in the data available to them. Pertinent to the clinical outcomes under the study, information was not available regarding proteinuria, BP control, use of disease-modifying therapies such as renin-angiotensin-aldosterone system inhibitors, and strategies to reduce cardiovascular risk. An important consideration in the cross-sectional association between social determinants of health and CKD severity is the potential for reverse causation. For example, patients may not be able to exercise as often as they would like as a consequence of their CKD.

Irrespective of potential problems with the interpretation of study findings, there is a broader question to be asked: what defines success or failure of a multidisciplinary CKD clinic? If a clinic enrolls a patient with already advanced CKD, it is unlikely that any of the interventions used in the clinic will prevent that individual from developing kidney failure. However, if the goal of the clinic model was to provide education and support for the patient such that they felt confident in their decision to pursue a specific dialysis modality or a kidney transplant, would we not deem that a success? It has been shown time and again that patient priorities do not necessarily align with those of their health care providers. International focus groups have demonstrated that, although patients with CKD place a high value on maintaining their kidney health, both patients and their caregivers prioritize other outcomes which are not typically captured in CKD registries such as life participation and mental health.^7^ Attempts to improve these outcomes for patients certainly demands input from a diverse group of providers as part of a holistic model of care, but in the absence of quantifying them, it is inherently difficult to recognize the value of that care and advocate for the resources needed to provide it within the clinic infrastructure. This is important because the day-to-day running of a multidisciplinary clinic is expensive. Financial and human resources are not limitless in any health care system, and the decision to invest in a new clinic model represents an opportunity cost from not investing in other interventions. If we take the financial argument a step further, perhaps the paradigm should be flipped such that interdisciplinary teams are used to accelerate the implementation of therapies that can alter the course of the disease. The standard of care for patients with CKD is shifting all the time, owing to the incredible success of clinical trial programs for novel treatments that target different mechanisms of disease.^8^ Safe and effective implementation of multiple drugs which have different adverse effects will require robust protocols for drug initiation, monitoring, and dose escalation. This would seem to be the ideal setting for a multidisciplinary clinic model which can leverage pharmacy expertise, nurse-led management protocols, and a registry for tracking care processes and clinical outcomes.

We should not underestimate the importance of learning from the experiences of others, especially for the implementation of a new intervention. Even a successful clinic model may not translate into a different environment. Ultimately, the appropriate choice of care model and team structure needs to fit within the landscape of where patients are receiving their care. This encompasses considerations such as funding availability, at-risk populations including indigenous groups who may have challenges accessing care due to distance from the hospital or other resource constraints,^9^ and communication challenges in a multiethnic population. On a philosophical level, individual programs arguably need to first define what success would look like in terms of both promoting patient equity and meeting the specific needs of the populations they serve.
